# Detection of circulating hybrid cells and circulating tumor cells in cerebrospinal fluid for leptomeningeal metastasis diagnosis and therapeutic prediction

**DOI:** 10.3389/fcell.2025.1716168

**Published:** 2025-12-01

**Authors:** Yanting Liu, Jiaxin Liang, Min Zhang, Nianhua Cao, Jie Xue, Xiaodi Zhang, YongXu Jia, Zhiwei Chang, Guodong Zhang, Yan Li, Hongyan Hui, Ruijuan Fan, Kelei Zhao, Zongbin Liu, Guifang Zhang, Ping Lu, Yinghua Ji

**Affiliations:** 1 Department of Oncology, The First Affiliated Hospital of Xinxiang Medical University, Weihui, Henan, China; 2 Department of Oncology, Xinxiang Central Hospital, The Fourth Clinical College of Xinxiang Medical University, Xinxiang, Henan, China; 3 Shenzhen Zigzag Biotechnology Co., Ltd., Shenzhen, China; 4 Department of Oncology, The First Affiliated Hospital of Zhengzhou University, Zhengzhou, Henan, China; 5 Huaxian Hospital of The First Affiliated Hospital of Xinxiang Medical University, Huaxian, Henan, China; 6 Department of Clinical Pharmacy, The First Affiliated Hospital of Xinxiang Medical University, Weihui, Henan, China

**Keywords:** leptomeningeal metastasis, diagnosis, cerebrospinal fluid, circulating tumor cells, circulating hybrid cells

## Abstract

**Purpose:**

Leptomeningeal metastasis (LM) represents a serious complication of advanced malignancies with poor prognosis. Conventional diagnostic approaches, such as MRI and cerebrospinal fluid (CSF) cytology lack sufficient sensitivity—particularly in early-stage disease or when imaging modalities are inconclusive. This study explores the diagnostic utility of circulating tumor cells (CTCs) and circulating hybrid cells (CHCs) in CSF for LM detection.

**Methods:**

A cascaded filter deterministic lateral displacement microfluidic chip was utilized to enrich CTCs and CHCs from the CSF of 27 patients with LM and 22 individuals with non-neoplastic neurological conditions. Identification of CSF-derived CTCs and CHCs was based on immunofluorescence staining combined with characteristic neoplastic morphology.

**Results:**

The detection frequency and absolute counts of CTCs and CHCs were markedly elevated in LM patients compared to non-LM controls (p < 0.0001). A positive correlation was observed between CTC and CHC levels (rs = 0.8406, p < 0.0001). ROC analysis revealed robust diagnostic performance, with AUC values of 0.8727 for CTCs, 0.8600 for CHCs, and 0.9545 for CK-positive cells (a composite of CTCs and CHCs). Importantly, in two LM cases where MRI and cytology failed to provide diagnostic confirmation, CSF-CTC and CHC analyses successfully identified significant cell counts.

**Conclusion:**

CSF-CTCs and CHCs represent promising biomarkers for LM diagnosis and therapeutic prediction with high sensitivity and specificity, complementing traditional MRI and cytology diagnostic approaches. These findings highlight their potential clinical utility and underscore the need for further studies to explore CHC formation mechanisms and their implications in LM pathogenesis and treatment strategies.

## Key Points

- CHCs and CTCs substantially exist in neoplastic CSF
- CHCs and CTCs complement MRI and CSF cytology in LM diagnosis
- CTCs and CHCs are promising biomarkers for monitoring ITC efficacy

### Importance of the study

Early diagnosis and monitoring of leptomeningeal metastases (LM) remain significant challenges. Previous studies have reported that cerebrospinal fluid circulating tumor cells (CSF-CTCs) exhibit higher diagnostic sensitivity compared to conventional CSF cytology. This study harnesses microfluidic CFD-Chips to achieve high-throughput and high-sensitivity detection of CSF-CTCs in LM patients. For the first time, we also report the abundant presence of circulating hybrid cells (CHCs) in the CSF of LM patients. This study demonstrates that CHCs and CTCs complement MRI and CSF cytology in the diagnosis of LM, particularly in early-stage LM or when imaging is not feasible. Furthermore, CHCs and CTCs are promising biomarkers for monitoring the efficacy of intrathecal chemotherapy (ITC), providing valuable insights into the understanding of LM biology and exploration of therapeutic targets.

## Introduction

Leptomeningeal metastasis (LM)—also referred to as leptomeningeal carcinomatosis or carcinomatous meningitis—is a devastating neurological manifestation of systemic malignancies, most commonly arising from lung cancer (LC), breast cancer (BC), and melanoma. Its incidence varies by cancer type, with estimates ranging from 5% to 20%, and appears to be increasing over time, as a result of improvements in the management and survival of systemic malignancies (Le Rhun et al., 2017; Remsik and Boire, 2024). LM leads to considerable morbidity and mortality, presenting with a wide spectrum of neurological symptoms such as headache, nausea, neurocognitive decline, cranial nerve deficits, gait disturbance, limb weakness, bowel and bladder dysfunction, altered consciousness, and seizures (Remsik and Boire, 2024; Freret and Boire, 2024; Nguyen et al., 2023).

Although multimodal therapies—including radiotherapy, chemotherapy, immunotherapy, targeted therapy, and surgical interventions—have been implemented in clinical practice upon diagnosis (Suh et al., 2020; Sener et al., 2021; Le Rhun et al., 2023; Beauchesne, 2010), the prognosis after the development of LM from most cancer types remains dismal, with median survival is limited to 6–8 weeks without tumor-specific treatment, as the appearance of the disease in the brain is frequently a hallmark of disseminated end-stage disease, whereas survival may be prolonged to a few months with LM-directed treatment (Le Rhun et al., 2023; Le Rhun et al., 2017; Le Rhun et al., 2019). This underscores the urgent need for sensitive and reliable diagnostic methods to enable early detection and prediction of response throughout the individualized management of LM (Le Rhun et al., 2017).

Early diagnosis of LM and disease monitoring remains challenging (Goldberg et al., 2024; Ruotolo et al., 2023; Jeffus et al., 2024). Thorough neurological examination is recommended but lack in sensitivity and specificity. Cerebrospinal fluid (CSF) cytology, though regarded as the gold standard for LM diagnosis due to its high specificity, suffers from low sensitivity, particularly in early disease stages, owing to the scarcity of malignant cells in CSF and technical limitations. As a result, repeat sampling is often necessary if the initial cytological analysis is negative (Ruotolo et al., 2023; Straathof et al., 1999; Bönig et al., 2019). The sensitivity and specificity of neuroimaging such as magnetic resonance imaging (MRI) or computed tomography (CT) remain difficult to appreciate due to a limited number of publications in patients with a suspicion of LM and improvement of technique over time, but have been estimated in the range of 66%–98% and 77%–97.5%, respectively. MRI can even yield normal results in patients with tumor cells in the CSF without measurable leptomeningeal lesions. Invasive leptomeningeal biopsy is technically challenging, carries potential risks, and is often impractical for patients with LM. Nevertheless, it may be considered when CSF cytology yields consistently negative results, in the absence of a known malignancy, or when clinical and imaging findings remain inconclusive and require diagnostic clarification to guide treatment decisions. Given that CSF is relatively accessible via lumbar puncture and contains tumor-derived components, it provides a valuable window into the biology of LM. Recently, CSF-based liquid biopsy has emerged as a promising, minimally invasive approach for detecting LM and tracking therapeutic responses. This includes analysis of circulating tumor cells (CTCs), cell-free tumor DNA (ctDNA), proteins, exosomes, and even nontumor immune-related cells (Goldberg et al., 2024; Wooster et al., 2022). However, the clinical application of such strategies is still in its infancy, with limited studies systematically evaluating their feasibility and clinical relevance in LM diagnosis and monitoring response (Roy-O’Reilly et al., 2023; Nakasu et al., 2023).

To initiate metastasis, cancer cells must detach from the primary tumor, survive the circulatory system, and colonize distant sites by establishing a supportive niche and evading immune surveillance. CTCs are tumor seeds disseminated through the bloodstream, which have currently been widely used to monitor tumor evolution and heterogeneity, as well as to detect treatment resistance, minimal residual disease, and recurrence in various cancers, including breast (Rack et al., 2014; Aceto et al., 2014; Vilalta et al., 2014; Pierga et al., 2008; Papadaki et al., 2020; Andre et al., 2022), lung (Tamminga et al., 2019; Zhu et al., 2021; Moon et al., 2020; Kanayama et al., 2022), and colorectal (Biller and Schrag, 2021; Li et al., 2023) malignancies (Ring et al., 2022; Labib and Kelley, 2021; Ahn et al., 2021; Palmela et al., 2021). The specific molecular mechanisms that enable cancers to effectively complete the typical process of leptomeningeal colonization, proliferation, and immune evasion remain elusive, an up-to-date research has indicated that BC may exploit neural signaling pathways for bone-to-meninges metastasis (Whiteley et al., 2024). An increasing number of studies have demonstrated circulating hybrid cells (CHCs) exhibiting both tumor and immune cell characteristics play important roles in tumor drug resistance, immune escape, metastasis and recurrence (Henn et al., 2021; Dietz et al., 2021; Manjunath et al., 2020; Chen et al., 2024). Recently, CSF-CTCs have been shown to have improved diagnostic performance for LM combined with magnetic resonance imaging (MRI) and CSF cytology. Nonetheless, whether CHCs are present in CSF and whether CSF-CHCs can serve as a liquid biomarker for LM diagnosis and therapeutic prediction remain to be clarified.

In this study, a cascaded filter deterministic lateral displacement microfluidic chip (CFD-Chip) (Liu et al., 2021) was harnessed to isolate CSF-CTCs and CSF-CHCs from CSF of 27 cancer patients with suspected LM. The CFD-Chip has been previously validated for its high efficiency, purity, and cell viability in enriching CTCs (Chen et al., 2024; Liu et al., 2021; Wang et al., 2024; Xu et al., 2025). LM was confirmed by CSF cytology or neuroimaging combined with clinical symptoms in 25 of the 27 patients, the remaining 2 patients, who were negative (or not feasible) for both CSF cytology and imaging, exhibited clinical symptoms consistent with LM and tested positive for CSF-CTCs and CSF-CHCs. Our results further highlight the clinical utility of CSF-CTCs and CSF-CHCs as non-invasive liquid biopsy biomarkers for LM diagnosis and for assessing responses to intrathecal chemotherapy (ITC), including agents such as pemetrexed, methotrexate, cytarabine, and dexamethasone. By providing dynamic monitoring of heterogeneous tumor cells in CSF, our finding revealed the significance of CSF-CTCs and CSF-CHCs in LM early diagnosis, facilitated improved prognostic predictions and tailored therapeutic strategies to individual patients, leading to better outcomes.

## Materials and methods

### Cell culture

The human breast cancer (BC) cell line MDA-MB-231 was cultured in Dulbecco’s Modified Eagle Medium (DMEM; CORNING, Cat#10-013-CVRC) supplemented with 10% fetal bovine serum (FBS; HyClone, Cat#SV30208.02) and 1% penicillin-streptomycin (HyClone, Cat#SV30010). The human lung cancer (LC) cell line A549 was maintained in RPMI-1640 medium (Basalmedia, Cat#L210KJ) containing 10% FBS and 1% penicillin-streptomycin. All cultures were incubated at 37 °C in a humidified atmosphere with 5% CO_2_ (ESCO, Singapore).

### Evaluation of capture efficiency

We employed Vybrant® Dye Cycle™ Green (Life Technologies, Carlsbad, CA) to label MDA-MB-231 or A549 cells, labeled cells were spiked into 5 mL cerebrospinal fluid (CSF) samples collected from patients, at concentrations of 20, 50, 100, 200, 500, and 1,000 cells. These samples were processed using the CFD-Chip designed by Zigzag Biotechnology (Shenzhen, China) (Chen et al., 2024; Liu et al., 2021; Wang et al., 2024; Xu et al., 2025), and recovered tumor cells were enumerated via immunofluorescence staining.

### Study design and patient sampling

This study was conducted in accordance with the ethical principles outlined in the Declaration of Helsinki. 18 LC patients, 7 BC patients, one gastric adenocarcinoma (GAC) patient, and one ovarian cancer (OC) patient presenting with symptoms associated with meningeal metastasis—including headache, nausea, vomiting, neurocognitive changes, gait difficulties, cranial nerve palsies, weakness, constipation, urinary retention, loss of consciousness, and seizures—were consecutively enrolled at The First Affiliated Hospital of Xinxiang Medical University, Xinxiang Central Hospital, and The First Affiliated Hospital of Zhengzhou University between January 2021 and December 2024. Additionally, 22 patients with non-neoplastic neurological disorders were included as controls, encompassing cases of ruptured intracranial aneurysms with subarachnoid hemorrhage, central nervous system (CNS) infections, hydrocephalus, intraventricular hemorrhage, cerebral infarction, and Guillain-Barré syndrome.

All 26 tumor patients underwent MRI examinations, while one LC patient was deemed unsuitable for MRI due to physiological condition. Three tumor patients did not undergo CSF cytology testing since MRI suggesting LM. The remaining 24 tumor patients had CSF cytology testing performed.

This study was approved by the Ethics Committee of The First Affiliated Hospital of Xinxiang Medical University. Written informed consent was obtained from all patients or their legal guardians prior to participation. Cerebrospinal fluid (CSF) samples (5 mL each) were collected into EDTA-containing tubes, maintained at room temperature, and processed within 4 h of collection to ensure sample integrity. Clinical information—including sex, age, cancer type, and Karnofsky Performance Status (KPS) scores—for all enrolled patients is summarized in Table 1.

**TABLE 1:** 25 LM patients’ clinical characteristics.

Characteristic	N	CTC positive (ratio)	CTC count (median [range])	P Value	CHC positive (ratio)	CHC count (median [range])	P Value	(CTC & CHC) positive (ratio)	(CTC & CHC) count (median [range])	P Value
Total	25	19 (76.00%)	16 [0, 20,000]	—	18 (72.00%)	2 [0, 98]	—	23 (92.00%)	16 [0, 20,098]	—
Sex										
Male	5	3 (60.00%)	1 [0, 28]	0.1223	3 (60.00%)	1 [0, 1]	**0.0479**	5 (100.00%)	2 [1, 28]	0.0938
Female	20	16 (80.00%)	16.5 [0, 20,000]		15 (75.00%)	4 [0, 98]		18 (90.00%)	25 [0, 20,098]	
Age (median = 56)										
<median	12	9 (75.00%)	22 [0, 3,400]	0.7571	6 (50%)	1 [0, 50]	0.2938	10 (83.33%)	29.5 [0, 3,400]	0.7794
≥ median	13	10 (76.92%)	6 [0, 20,000]		12 (92.31%)	4 [0, 98]		13 (100.00%)	11 [1, 20,098]	
Cancer										
LC	16	13 (81.25%)	6 [0, 730]	0.2817	12 (75.00%)	1.5 [0, 25]	0.3426	16 (100.00%)	13 [1, 736]	0.1487
BC	7	5 (71.43%)	39 [0, 3,400]		5 (71.43%)	5 [0, 50]		6 (85.71%)	89 [0, 3,400]	
GAC	1	1 (100.00%)	20,000		1 (100.00%)	98		1 (100.00%)	20,098	
OC	1	0 (0.00%)	0		0 (0.00%)	0		0 (0.00%)	0	
KPS										
<80%	11	9 (81.82%)	6 [0, 20,000]	0.8604	9 (81.82%)	3 [0, 98]	0.4354	10 (90.91%)	15 [0, 20,098]	0.7776
≥80%	14	10 (71.43%)	22 [0, 3,400]		9 (64.29%)	1 [0, 50]		13 (92.86%)	22 [0, 3,400]	

A statistically significant difference in CHC counts was observed between males and females (p = 0.0479), suggesting a potential sex-related difference that should be further evaluated in larger clinical studies.

### CSF sample processing

Five milliliters of CSF from each participant were diluted with an equal volume of phosphate-buffered saline (PBS) and processed using the CFD-Chip (Chen et al., 2024; Liu et al., 2021; Wang et al., 2024; Xu et al., 2025). The CFD-Chip employs a hydrodynamic cell-sorting design that integrates a size-based filtration concept into a deterministic lateral displacement structure. This cascaded microfluidic configuration enables high-throughput, clog-free isolation of rare cells with enhanced performance in size-based separation, achieving high capture efficiency, purity, cell viability, and processing rate. The processing time for each sample was approximately 25 min. Collected cell suspensions were centrifuged at 250 *g* for 10 min and were resuspended in PBS prior to analysis.

### Immunofluorescence

After enrichment, the cell suspension was plated into 96-well plates and fixed with 4% paraformaldehyde (Affymetrix, Santa Clara, CA) for 15 min at room temperature. Cells were then washed with PBS and permeabilized using 0.1% Triton X-100 (Sigma-Aldrich, St. Louis, MO) for 10 min. Following a 1-h blocking step with 3% bovine serum albumin (BSA), cells were incubated overnight at 4 °C with a primary antibody cocktail: anti-CD45-PE (1:100, clone HI30, Cat#555483, BD Biosciences, RRID: AB_395875) and anti-pan-cytokeratin-Alexa Fluor™ 488 (1:500, clone AE1/AE3, Cat#53-9003-82, Thermo Fisher, RRID: AB_1834350). Following six washes, cells were incubated with 15 μL of DAPI solution (1 mg/mL; Solarbio, Cat# C0060, Beijing, China) for 10 min. Subsequently, cells were rinsed twice with PBS and examined under a fluorescence microscope.

### Identification of CSF-CTCs and CSF-CHCs

We adopted the following morphological and immunophenotypic criteria to define malignant cells. First, tumor-like cells exhibited irregular, pleomorphic nuclei with a high nuclear-to-cytoplasmic ratio and prominent or multiple nucleoli. Second, cells showing strong cytokeratin expression and tumor-like nuclear morphology were classified as malignant. Cells with nonspecific fluorescence adsorption or lacking tumor-like nuclear features were excluded. Specifically, CSF-CTCs were defined as DAPI^+^/CD45^-^/CK^+^ cells with tumor-like nuclear morphology and intact membranes. CSF-CHCs were defined as DAPI^+^/CD45^+^/CK^+^ cells showing heterogeneous membrane features but also containing a tumor-like nucleus.

All images were independently reviewed by two trained cytopathologists blinded to clinical data. Although inter-observer agreement (kappa) was not statistically assessed, any discrepancies were resolved by consensus review.

### Statistical analysis

Data analysis was carried out using GraphPad Prism 10 and IBM SPSS Statistics 26. Data are presented as boxplots displaying all individual values; boxes represent interquartile ranges, horizontal lines indicate medians, and whiskers denote the full data range. Group comparisons for CTC and CHC counts were performed using the Mann-Whitney test due to CTC and CHC counts fit non-Gaussian distribution. The association between CTC and CHC numbers was confirmed by Spearman correlation test. Receiver operating characteristic (ROC) curve analyses were performed using GraphPad Prism version 10 to evaluate the diagnostic performance of CTCs and CHCs for LM. In Prism, the area under the ROC curve (AUC) and its 95% confidence interval are calculated using the nonparametric method of Hanley and McNeil, which analytically estimates the standard error and applies the normal (z) approximation for CI construction. The positivity threshold “>0.5 cells per 5 mL” indicates that ≥1 cell per 5 mL CSF is considered positive. A two-tailed p-value was considered statistically significant if ≤ 0.05.

## Results

### Tumor cell capture efficiency of CFD-Chip

**FIGURE 1:**
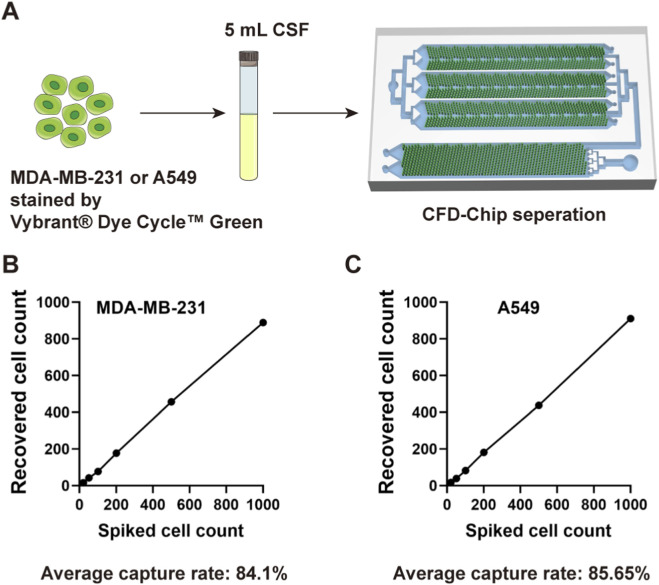
The CFD-Chip capture efficiency. **(A)**, MDA-MB-231 or A549 cells were used to confirm the robust capture efficiency of the CFD-Chip for breast and lung cancer. **(B,C)**, The capture efficiency of MDA-MB-231 **(B)** and A549 **(C)** tumor cells in the CSF samples by the CFD-Chip.

MDA-MB-231 or A549 cells were labeled by Vybrant® Dye Cycle™ Green, spiked into 5 mL CSF specimens and then perfused into CFD-Chip at cell counts of 20, 50, 100, 200, 500, and 1,000, respectively (Figure 1A). The CFD-Chip exhibited reliable separation efficiency for tumor cells in CSF, achieving average recovery rates of 84.1% and 85.65% for MDA-MB-231 and A549 cells, respectively. Correlation curves in Figures 1B,C depicted the recovery rate and spiked cell count detailly.

### Study design and detection of CTCs and CHCs in CSF

**FIGURE 2:**
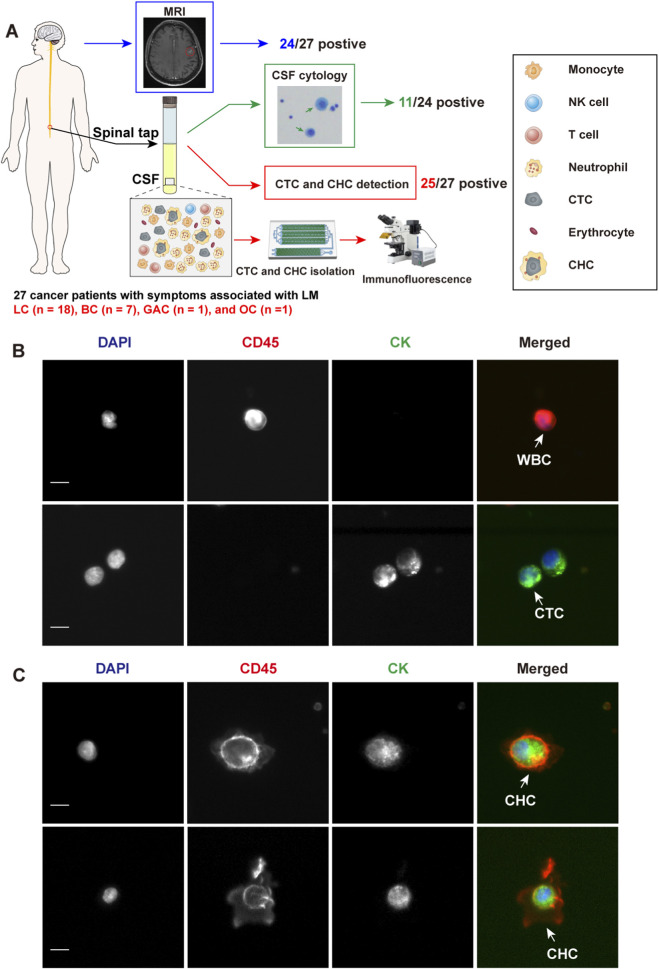
Study design and identification of circulating tumor cells (CTCs) and circulating hybrid cells (CHCs) in cerebrospinal fluid (CSF) by immunofluorescence. **(A)**, Schematic diagram of the study design. MRI and CSF cytology are employed to diagnosis leptomeningeal metastasis (LM) if feasible. CFD-Chip was used to isolate CTCs and CTC clusters from CSF. **(B)**, White blood cells (WBCs) were detected by DAPI+/CD45+/CK- immunofluorescence staining. CTCs were detected by DAPI+/CD45-/CK + immunofluorescence staining. Scale bar, 10 µm. **(C)**, CHCs were detected by DAPI+/CD45+/CK + immunofluorescence staining. Scale bar, 10 µm.

27 neoplastic patients with LC (n = 18), BC (n = 7), GAC (n = 1), and OC (n = 1) presenting with symptoms suggestive of LM, were consecutively enrolled to assess the clinical significance of CTCs and CHCs in LM diagnosis (Figure 2A). The gold standard for LM diagnosis is CSF cytology; nonetheless, due to its low sensitivity, the diagnostic criteria for LM in this study were MRI positivity along with LM-related clinical symptoms, including headache, nausea, vomiting, neurocognitive changes, gait difficulties, cranial nerve palsies, weakness, constipation, urinary retention, loss of consciousness, and seizures. Among the 27 enrolled patients, 24 had MRI findings consistent with meningeal metastasis (Figure 2A). Of the remaining three patients, two had no abnormal meningeal thickening on the MRI, and the other was unable to undergo an effective MRI scan due to the patient’s condition. 24 of the 27 patients underwent CSF cytology, which was performed using Wright-Giemsa staining; however, only 11 patients had tumor cells identified in the CSF (Figure 2A). The clinical characteristics of the 25 patients with confirmed LM are summarized in Table 1.

All 27 patients underwent CTC and CHC analyses of their CSF, with CTCs or CHCs identified in the CSF of 25 patients. The enumeration of CTCs was performed manually, based on cells exhibiting DAPI+/CD45-/CK + immunofluorescence staining and distinct neoplastic morphology (Figure 2B). Similarly, CHC counts were determined by identifying DAPI+/CD45+/CK + cells with intact neoplastic morphology (Figure 2C). Intriguingly, CHCs displayed unique morphological features, highlighting the need for further investigation into the mechanisms of CHC formation in CSF. Such studies could provide critical insights into the immune microenvironment of LM.

The summarizations of CTC and CHC count in 25 patients diagnosed with LM by MRI or CSF cytology were outlined in Table 1. CTCs or CHCs were detected in the CSF of 23 LM patients, whereas one patient with invasive ductal carcinoma of the breast and the OC patient showed no detectable CTCs or CHCs in the CSF. CSF cytology also failed to detect any tumor cells in these two patients while LM was diagnosed by MRI. Statistical analysis revealed no significant correlation between the number of CTCs or CHCs and patient age or KPS scores (p > 0.05). Due to the relatively small sample size, no association was observed between CTC/CHC counts and cancer type (p > 0.05), potentially because most LM patients were in advanced stages of the disease (Table 1). Intriguingly, female patients with LM may exhibit a higher CHC count compared to male patients (p = 0.0479) while no significant difference was observed in CTC count. Of note, in the patient with GAC, 20,000 CTCs and 98 CHCs were detected in 5 mL of CSF, while no CTCs or CHCs were found in the OC patient (Table 1).

### Detection of CTCs and CHCs in CSF for LM diagnosis

**FIGURE 3:**
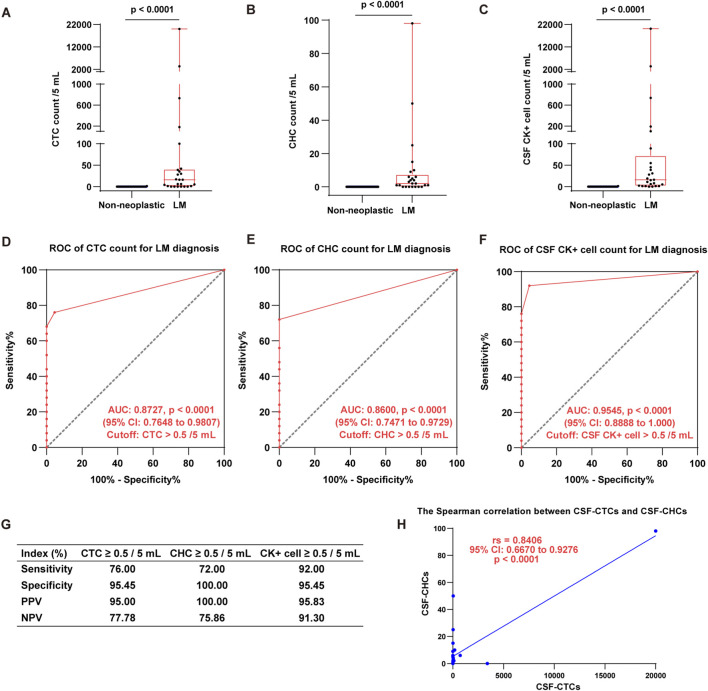
Detection of CTCs, CHCs, and CK + cells in CSF for LM diagnosis. **(A–C)**, The boxplots show CTC **(A)**, CHC **(B)**, and CK + cell (CTC & CHC) **(C)** counts per 5 mL CSF in LM patients compared to those with non-neoplastic neurological disorders (Mann-Whitney test, p < 0.0001). **(D–F)**, The ROC analyses demonstrate the diagnostic utility of CSF-CTCs **(D)**, CSF-CHCs **(E)**, and CSF CK + cell **(F)** counts for LM diagnosis. The AUC and optimal cutoff are indicated in the graphs. **(G)**, The table summarizes the diagnostic indices for each biomarker, including sensitivity, specificity, positive predictive value (PPV), and negative predictive value (NPV). These results underscore the high diagnostic accuracy of CTC, CHC, and CK + cell counts for LM diagnosis. **(H)**, There is a significant positive correlation between the number of CSF-CTC and CSF-CHC count (Spearman test, p = 0.0004). (“>0.5 cells per 5 mL” indicates that ≥1 cell per 5 mL CSF is considered positive).

The positive rates and counts of CTCs and CHCs in the CSF of 25 cancer patients with LM were significantly elevated compared to 22 patients with non-neoplastic neurological disorders (p < 0.0001). CTCs were detected in 19 of the 25 LM patients, corresponding to a positive rate of 76.00%. The CTC counts ranged from 0 to 20,000 with a median count of 16 per 5 mL. In comparison, only one CTC was detected in a single patient among the 22 with non-neoplastic neurological disorders (Figure 3A). Similarly, CHCs were identified in 18 of the 25 LM patients, resulting in a positive rate of 72.00%. The CHC counts ranged from 0 to 98 per 5 mL, with a median count of 2 per 5 mL. In contrast, no CHCs were found in any of the 22 patients with non-neoplastic neurological disorders (Figure 3B). Furthermore, CK + cells (the combined total of CTCs and CHCs) were detected in the CSF of 23 out of the 25 LM patients, achieving a positive rate of 92.00% (Figure 3C).

We further evaluated the diagnostic potential of CTCs and CHCs for LM using receiver operating characteristic (ROC) curve analysis. The results demonstrated that both CTCs and CHCs are promising diagnostic markers for LM, with high predictive specificity. The area under the ROC curve (AUC) for CTCs was 0.8727 (95% CI: 0.7648 to 0.9807, p < 0.0001) (Figure 3D). The optimal diagnostic threshold, determined by the maximum Youden index, was CTC >0.5 per 5 mL, with sensitivity and specificity of 76.00% and 95.45%, respectively. The positive predictive value (PPV) and negative predictive value (NPV) were 95.00% and 77.78%, respectively (Figure 3G). For CHCs, the ROC analysis yielded an AUC of 0.8600 (95% CI: 0.7471 to 0.9729, p < 0.0001), with an optimal cutoff of CHC >0.5 per 5 mL. This threshold demonstrated a sensitivity of 72.00% and a specificity of 100.00% (Figure 3E). Additionally, ROC analysis for CK + cells (the combined total of CTCs and CHCs) revealed an AUC of 0.9545 (95% CI: 0.8888 to 1.000, p < 0.0001). At a cutoff of CK + cells >0.5 per 5 mL, the sensitivity and specificity were 92.00% and 95.45%, respectively (Figure 3F). (“>0.5 cells per 5 mL” indicates that ≥1 cell per 5 mL CSF is considered positive).

Furthermore, we employed the Spearman rank correlation to evaluate the relationship between CTC and CHC counts in the CSF. The analysis revealed a strong positive correlation between CTC and CHC counts (rs = 0.8406, 95% CI: 0.6670 to 0.9276, p < 0.0001) (Figure 3H). These findings underscore the diagnostic value of CTC, CHC, and CK + cell counts in detecting LM, highlighting their potential as reliable biomarkers for clinical application. The diagnostic indices for each biomarker, including sensitivity, specificity, PPV, and NPV, are summarized in Figure 3G.

### CTC and CHC detection may be complementary to imaging and CSF cytology detection in LM diagnosis

MRI is an effective diagnostic tool for LM. However, its sensitivity may be limited in certain cases, and it may fail to provide reliable detection under specific circumstances. Among 27 enrolled patients presenting with symptoms indicative of LM, one lung cancer patient (#23520930) showed no evidence of abnormal meningeal thickening or LM-related indicators on MRI, while another patient (#23535856) was unable to undergo a diagnostic MRI scan due to their clinical condition. Additionally, CSF cytology for both patients did not detect any tumor cells. However, CSF-CTC and CSF-CHC analyses revealed findings suggestive of LM. Specifically, patient #23520930 had 5,000 CTCs and 5 CHCs detected in 5 mL of CSF, while patient #23535856 had 73 CTCs and 72 CHCs in 5 mL of CSF (Table 2). These results highlight the complementary role of CTC and CHC detection in LM diagnosis, particularly in identifying LM at early stages that may be missed by MRI or in patients for whom MRI is not feasible.

**TABLE 2:** Putative CTC and CHC detection in CSF indicated potential LM in two LC patients.

Patient	MRI	CSF cytology	CTCs/5 mL	CHCs/5 mL
#23520930	Negative 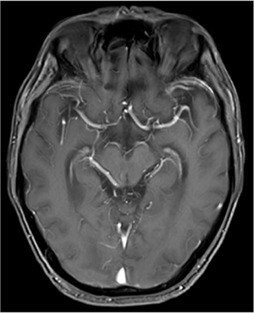	Negative 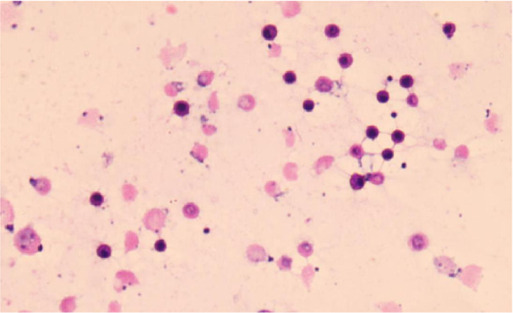	5,000	5
#23535856	Not feasible	Negative 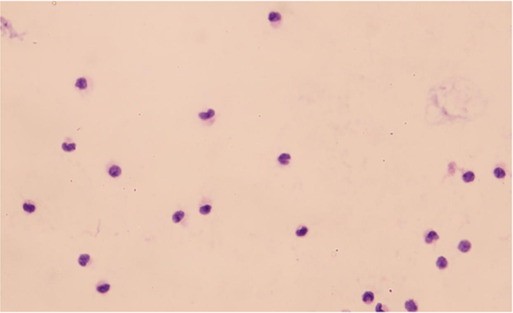	73	72

### CTC and CHC detection for dynamic efficacy assessment of intrathecal chemotherapy

ITC is currently the mainstay treatment for patients with LM, but its efficacy is difficult to monitor dynamically, and drug resistance often occurs (Beauchesne, 2010). Dynamic efficacy monitoring and the timely detection of potential resistance are crucial for adjusting chemotherapy regimens and achieving personalized treatment, which is essential for improving the survival and quality of life of LM patients. Conventional methods, such as MRI and CSF cytology, have limitations due to insufficient sensitivity for dynamic assessment of ITC efficacy (Beauchesne, 2010; Le Rhun et al., 2019; Salimian et al., 2024).

**FIGURE 4:**
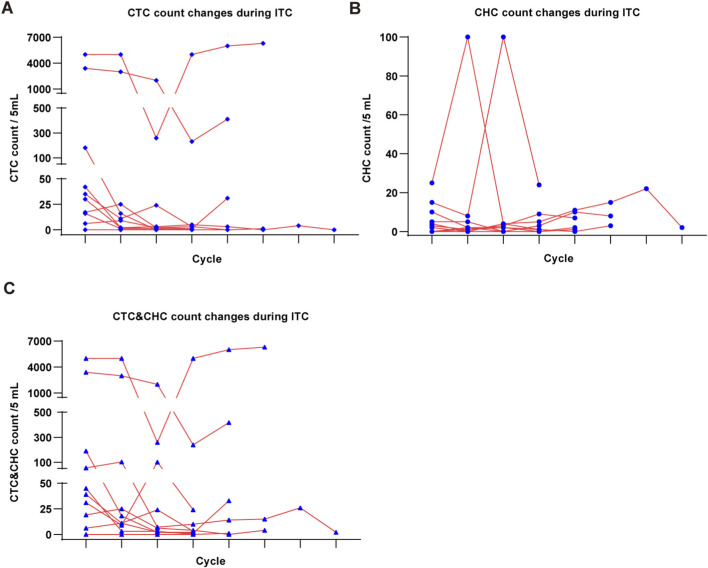
Dynamic monitoring of ITC efficacy by CSF-CTC and CSF-CHC detection. **(A)**, The line chart shows the CTC count changes per 5 mL CSF in LM patients during ITC. **(B)**, The CHC count changes per 5 mL CSF during ITC. **(C)**, The CSF CK + cell (CTC&CHC) count changes per 5 mL CSF during ITC.

In this study, we performed longitudinal dynamic monitoring of CTC and CHC counts in 10 LM patients who underwent ITC, with at least four cycles of monitoring, to evaluate the potential of CTC and CHC detection for assessing ITC efficacy. Our results demonstrated that the CSF-CTC count generally decreased during the initial treatment cycles in most patients, suggesting effective suppression of neoplastic meningitis by ITC (Figure 4A). However, we observed fluctuating increases in the CSF-CHC count throughout the treatment, which may indicate the possible evolution of resistance to ITC in LM, suggesting an adaptive response of the tumor to therapy. This fluctuation could reflect the early stages of treatment resistance (Figure 4B). In addition, the overall CSF CK + cell (CTC&CHC) count decreased during the early cycles of treatment but later rebounded in subsequent cycles. This rebound could signify the development of drug resistance to the initial ITC regimen, suggesting that tumor progression and resistance may be occurring (Figure 4C). These findings highlight the need for timely adjustments to the ITC regimen to enhance therapeutic efficacy and overcome emerging resistance.

## Disscussion

LM presents significant diagnostic and therapeutic challenges due to its rapid progression and nonspecific clinical manifestations. Although the natural history of LM remains incompletely understood, most patients experience a brief course from the onset of symptoms to death (Remsik and Boire, 2024; Freret and Boire, 2024). While LM is typically associated with the terminal stage of widely disseminated systemic cancers, approximately 10% of cases manifest as the initial presentation of malignancy (Goldberg et al., 2024).

The diagnostic evaluation of LM relies heavily on MRI with contrast as the imaging modality of choice. However, conventional MRI has limitations in sensitivity, particularly in early-stage LM or cases where meningeal thickening is minimal or absent. Cytological assessment of CSF is currently considered the gold standard for LM diagnosis, offering a high specificity of up to 100% (Goldberg et al., 2024). However, its sensitivity is notably limited due to the low concentration of tumor cells in the CSF, which are often significantly outnumbered by immune cells. This sparse presence of tumor cells increases the likelihood of false-negative results, particularly in early-stage LM or when CSF samples are not optimally processed (Nguyen et al., 2023).

Recent advancements highlight the potential of CSF-CTCs as promising biomarkers for LM diagnosis and monitoring. The methodologies employed in CSF-CTC isolation predominantly include CellSearch® assay and flow cytometry (Acosta et al., 2016; Milojkovic et al., 2015; Subirá et al., 2011; Diaz et al., 2022). Studies have reported the diagnostic utility of CSF-CTCs in various cancers, including lung, breast, melanoma, ovarian, and gastrointestinal cancers (Wooster et al., 2022; Barbour et al., 2024; Muddasani et al., 2024; Li et al., 2022). Compared to conventional cytology, CSF-CTC detection exhibits superior sensitivity and comparable specificity, with additional benefits such as correlation with tumor burden, functional status, and response to therapy (Milojkovic et al., 2015; Wijetunga et al., 2021; van Bussel et al., 2020; Malani et al., 2020). Importantly, CSF-CTC counts have been shown to predict survival outcomes, offering a novel prognostic indicator in LM (Diaz et al., 2022). These findings underscore their potential not only as early diagnostic biomarkers but also as robust tools for assessing treatment response and monitoring disease dynamics (Nayak et al., 2013; Boire et al., 2019).

Despite these advancements, significant challenges remain, particularly due to the inherent heterogeneity of tumors. Tumor dissemination often involves diverse subpopulations of cells with unique characteristics, reflecting this heterogeneity. To the best of our knowledge, the presence of CHCs in CSF has not been documented to date. This absence may stem from the methodologies employed in prior studies, which predominantly relied on red blood cell lysis and subsequent antigen-antibody capture techniques. Such methods are limited in isolating cells with dual phenotypes—those exhibiting both immune and tumor cell characteristics—thereby likely overlooking CHCs in the CSF. In contrast, CHCs have been identified in peripheral blood, where they have been implicated in critical processes such as metastasis, therapeutic resistance, and survival prognosis (Henn et al., 2021; Dietz et al., 2021; Chen et al., 2024; Chou et al., 2023; Walker et al., 2021). These findings suggest that CHCs in the CSF could play a similarly pivotal role in LM. Investigating their presence and functional implications may provide valuable insights into the mechanisms of LM progression, the dynamics of its immune microenvironment, and potential biomarkers for diagnosis or therapeutic intervention.

In this study, we utilized the CFD-Chip to efficiently capture CTCs and, for the first time, detect CHCs in the CSF. The significantly higher detection rates and counts of CTCs and CHCs in LM patients compared to those with non-neoplastic neurological disorders (p < 0.0001) highlight their specificity and sensitivity for LM diagnosis. ROC analysis further supports the diagnostic utility of these biomarkers, with the combined total of CTCs and CHCs showing the highest area under the curve (AUC) of 0.9545, followed by CTCs alone (AUC = 0.8727) and CHCs alone (AUC = 0.8600). Importantly, none of the 22 patients with non-tumor-related neurological disorders showed detectable CHCs. These results indicate excellent diagnostic performance when CTCs and CHCs are analyzed together for LM diagnosis.

Moreover, our study also highlights two critical cases where CSF-CTC and CHC analyses identified LM despite negative MRI and cytology findings. In patient #23520930, 5,000 CTCs and 5 CHCs were detected in 5 mL of CSF, while patient #23535856 exhibited 73 CTCs and 72 CHCs in 5 mL of CSF. These findings illustrate the potential of CSF-CTC and CHC analyses to detect LM in challenging diagnostic scenarios, offering a valuable tool for timely intervention and management, suggesting that CSF-CTC and CHC analyses can complement MRI and cytology, particularly in early-stage LM or in cases where imaging is not feasible due to patient conditions. Notably, the strong correlation between CTC and CHC counts (rs = 0.8406, p < 0.0001) suggests a potential mechanistic link in their formation and roles in LM progression. This interplay warrants further investigation, as it may provide novel insights into the LM microenvironment and the pathophysiology of tumor dissemination into the CNS.

Additionally, we demonstrate CTC and CHC detections hold significant promise for efficacy assessment in ITC, offering an important tool for early diagnosis and intervention. Aggressive treatment of low-risk LM patients is critical to preventing progressive neurological damage, underscoring the importance of personalized treatment strategies to optimize patient survival and quality of life (Le Rhun et al., 2017; Beauchesne, 2010; Le Rhun et al., 2019). The dynamic changes in CSF-CTC and CSF-CHC counts provide a non-invasive, real-time method to monitor disease progression and therapeutic response. These markers complement traditional diagnostic methods such as MRI and CSF cytology, which have limited sensitivity in assessing dynamic changes during treatment (Goldberg et al., 2024; Roy-O’Reilly et al., 2023; Salimian et al., 2024). Integrating CTC and CHC monitoring into clinical practice can help clinicians tailor treatment strategies more effectively, detect early signs of treatment failure, and potentially improve patient outcomes in this challenging and often fatal condition (Beauchesne, 2010).

We acknowledge certain limitations that warrant further exploration. First, the relatively small sample size limits the statistical robustness needed to comprehensively explore the associations between CSF-CTC/CHC counts and clinical parameters, such as tumor type, disease stage, epigenetic factors, and prognosis. Future studies with larger cohorts are essential to validate and extend these findings.

Second, while our study highlights the diagnostic utility of CSF-CTC and CHC analyses, integrating complementary biomarkers, such as cell-free DNA (cfDNA) extracted from CSF, could further refine diagnostic approaches. CSF-cfDNA carrying genetic alterations, chromosomal variations, and hypermethylation have been reported to hold significant clinical value in genomic profiling, targeted detection of driver mutations, and therapy monitoring (Fit et al., 2022; White et al., 2021; Choi et al., 2021; Chiang et al., 2021; Ma et al., 2020; De Mattos-Arruda et al., 2015; Ballester et al., 2018; Ying et al., 2019). Nonetheless, CSF-CTCs and CHCs offer unique advantages compared to cfDNA. As intact biological entities, they provide dynamic, multi-omics insights, encompassing protein, RNA, and DNA information. Moreover, they can be cultured *in vitro* to establish CTC lines, facilitating deeper investigations into metastatic mechanisms and potential therapeutic interventions. The integration of CSF-CTC and CSF-CHC analyses with CSF-cfDNA could improve the sensitivity and specificity of LM diagnosis, particularly in cases with minimal tumor cell shedding or when imaging modalities are not feasible. Such a biomarker integration approach could also uncover therapeutic implications, paving the way for personalized treatment strategies for LM patients.

Additionally, the mechanisms underlying CHC formation remain incompletely understood but are pivotal for understanding tumor-host interactions within the LM microenvironment. CHCs, which exhibit features of both tumor cell (CK+) and immune cell (CD45^+^), likely play a significant role in the immune evasion strategies employed by tumors within the CNS and are involved in the development of resistance to ITC. Recent research from our group indicates that CHCs in peripheral blood may originate from interactions between neutrophils and tumor cells. This interaction is demonstrated to endow these hybrid cells with enhanced survival traits, facilitating hematogenous dissemination (Chen et al., 2024). To further elucidate the formation mechanisms of CSF-CHCs, advanced omics techniques, including single-cell sequencing, are essential. These methods could uncover differentially expressed genes, reveal critical genetic drivers, and identify unique molecular abnormalities associated with LM. Such analyses would shed light on the interactions between tumor cells and CSF non-neoplastic cells, potentially leading to the emergence of CHCs and other hybrid phenotypes (Manjunath et al., 2020; Gast et al., 2018; Delespaul et al., 2020). Understanding these processes could be instrumental in developing therapeutic strategies targeting CHCs, ultimately contributing to novel and more targeted treatments for LM.

In conclusion, our study highlights the potential of CSF-CTCs and CHCs as promising diagnostic and efficacy-predictive biomarkers for LM, offering high sensitivity, specificity, and complementary utility to conventional methods such as MRI and cytology. These findings not only pave the way for incorporating these biomarkers into clinical practice but also provide new insights into the underlying mechanisms of LM pathogenesis and therapeutic resistance. Further studies with larger patient cohorts are essential to validate these findings and to broaden their clinical applicability. The integration of such advanced biomarker analyses into clinical workflows and investigating the mechanisms of CHC formation could significantly enhance the diagnostic and therapeutic landscape for the understanding of LM biology and identify novel therapeutic targets.

## Data Availability

The original contributions presented in the study are included in the article/supplementary material, further inquiries can be directed to the corresponding authors.
